# Snow flea antifreeze peptide for cryopreservation of lactic acid bacteria

**DOI:** 10.1038/s41538-022-00128-4

**Published:** 2022-02-03

**Authors:** Xu Chen, Jinhong Wu, Xiaozhen Li, Fujia Yang, Dan Huang, Jianlian Huang, Shaoyun Wang, Vincent Guyonnet

**Affiliations:** 1 College of Biological Science and Engineering, Fuzhou University, 350108 Fuzhou, Fujian China; 2 College of Chemical Engineering, Fuzhou University, 350108 Fuzhou, Fujian China; 3 Department of Food Science and Engineering, School of Agriculture and Biology, Shanghai Jiao Tong University, 200240 Shanghai, China; 4 Fujian Provincial Key Laboratory of Frozen Processed Aquatic Products, 361022 Xiamen, China; 5 Fujian Anjoy Food Co. Ltd, 361022 Xiamen, China; 6 FFI Consulting Ltd, 2488 Lyn Road, Brockville, ON K6V 5T3 Canada

**Keywords:** Expression systems, Peptides

## Abstract

Cryogenic machining is one of the most commonly used techniques for processing and preserving in food industry, and traditional antifreeze agents cannot regulate the mechanical stress damage caused by ice crystals formed during recrystallization or thawing. In this study, we successfully developed an express system of a novel recombinant snow flea antifreeze peptide (rsfAFP), which has significant ice recrystallization inhibition ability, thermal hysteresis activity and alters ice nucleation, thus regulating extracellular ice crystal morphology and recrystallization. We showed that rsfAFP improved the survival rate, acid-producing ability, freezing stability, and cellular metabolism activity of *Streptococcus thermophilus*. We further showed that rsfAFP interacts with the membrane and ice crystals to cover the outer layer of cells, forming a dense protective layer that maintains the physiological functions of *S. thermophilus* under freezing stress. These findings provide the scientific basis for using rsfAFP as an effective antifreeze agent for lactic acid bacteria cryopreservation or other frozen food.

## Introduction

Due to the high-water activity in fresh foods, chemical reactions and microbial growth are prone to occur, leading to food deterioration^1^. Cryopreservation is one of the most effective techniques for food storage^2^. However, frozen food continuously suffers from the damage caused by ice crystals growth or recrystallization, which damages the cell and tissue structure, so that weight loss, discoloration, protein denaturation, and nutrient loss of the products during freezing or freezing storage^3^. Moreover, cell-based frozen foods, such as freeze-dried probiotics are susceptible to mechanical damage caused by ice crystals, which eventually leads to reduced cell viability^4^. Therefore, an antifreeze agent is inevitably for frozen food to reduce mechanical damage caused by ice crystals during cryogenic machining or cryopreservation. At present, skim milk, sugars and phosphates are the most widely used antifreeze agents for the cryopreservation of food, while glycerol and dimethyl sulfoxide (DMSO) are typically used for cells^5^. Although very successful and widely used, these antifreeze agents are not the perfect solutions since they cannot regulate the formation of ice crystals during recrystallization or thawing. The mechanical stress damage of the ice crystals formed during recrystallization or thawing is even greater than during the freezing process^6^. In addition, traditional commercial antifreeze agents like sugar and phosphates will bring sweet taste, high salt content, and high calorie, while organic solvents like glycerol and DMSO, can potentially have cytotoxic effects and cause irreversible damage to cells^7^. Therefore, studies are needed to investigate innovative cryopreservation methods.

Antifreeze proteins (AFPs) are a unique class of proteins that enable organisms to survive in freezing and subfreezing environments^8^. Thermal hysteresis activity (THA), ice recrystallization inhibition (IRI) ability, and ice nucleation isomerization ability are key properties of AFPs^9–12^, making them promising novel cryoprotectants for application in frozen foods, cryobiology, or biological materials^10,13–16^. However, the large-scale applications of AFPs purified from natural organisms are difficult due to low yield and high purification costs^3^. Recently a glycine-rich AFP has been isolated from Canadian snow fleas^17^, and its molecular structure was reported by Pentelute et al.^18^ and Treviño et al.^19^. The snow flea AFP belongs to the family of hyperactive insect AFPs^20^, and its THA is 1–2 orders of magnitude higher than most AFPs found in plants and fish^21^, and could potentially be an excellent antifreeze agent for the cryopreservation of lactic acid bacteria (LAB)^22^. In the past, the research on snow flea AFP mainly focused on the interaction mechanism with ice crystals^23,24^. However, the protective mechanism of AFP on microorganisms under freezing stress is rarely studied.

Currently, the expression of heterologous proteins in bacteria is one of the main methods to overcome the low yield and high purification costs for the production of natural proteins. *B. subtilis* is a non-pathogenic microorganism with high safety and can be used in the food and medicine industry^25^. However, to our knowledge, there have been no reports about the expression and production of hyperactive snow flea AFP in *Bacillus subtilis*. Moreover, the cryoprotective mechanism of snow flea AFP on microorganisms is still unclear. Therefore, the present study attempts to express and produce a recombinant snow flea antifreeze peptide (rsfAFP) using *B. subtilis* (WB800N). The effects of rsfAFP on ice crystals morphology, TH, and IRI were investigated. Furthermore, the cryoprotective effect of rsfAFP on the physiological functions of *Streptococcus thermophilus* under freezing stress, and the mechanism by which rsfAFP interacts with these cells and ice were also studied. Findings from this work will provide new insights into the mechanism by which rsfAFP regulates some cell physiological functions and the formation of ice crystals under freezing stress. In addition, rsfAFP as an antifreeze agent has great potential value in food processing and storage.

## Results

### Expression, purification, and identification of rsfAFP

**Fig. 1:**
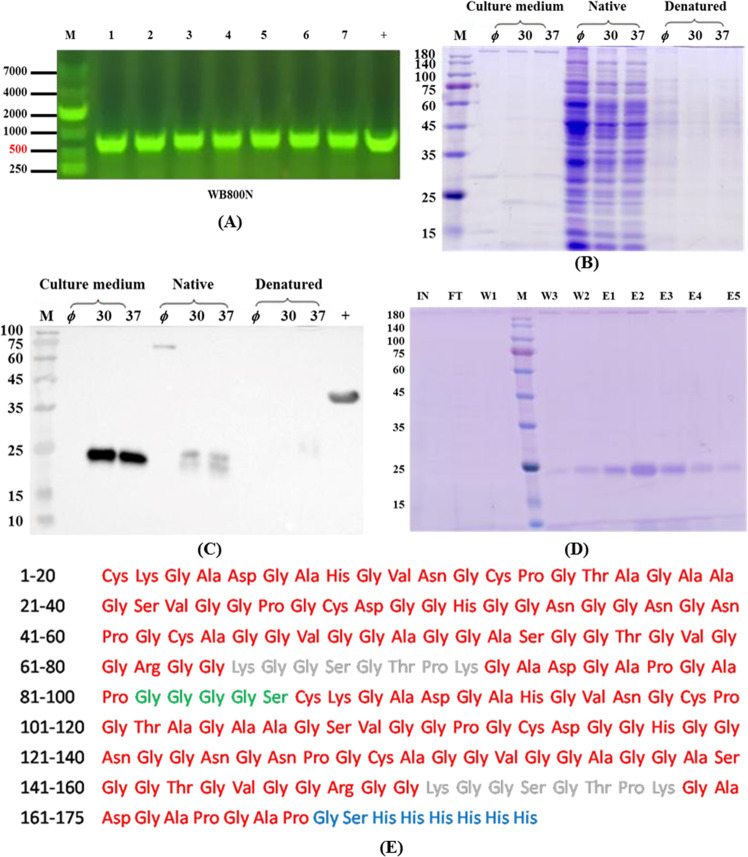
Expression and identification of rsfAFP in *B. subtilis* WB800N. **A** PCR detects positive clones. M: DNA marker DL10000; lanes 1–7: recombinant plasmid; +: positive control. **B** Expression result. Coomassie blue staining. MW: molecular weight marker. *ϕ*: Transformed cells not induced. **C** Western blot detects expression results. MW: molecular weight marker. *ϕ*: transformed cells not induced. +: positive control. **D** Target protein purification profile. IN: input; FT: flow through; MW: molecular weight marker; W1– W3: wash with TBS buffer with 0, 30, and 50 mM imidazole, respectively; E1–E5: eluted with TBS buffer with 100, 150, 200, 250, and 400 mM imidazole, respectively. **E** Sequence cover situation of rsfAFP by Nano LC-MS/MS spectrometry. The red section indicates the peptides matching the peptides of the snow flea antifreeze protein. The gray section indicates the missing fragments in the Nano LC-MS/MS test. The green section of peptide is GS linker. The blue section of peptide is 6*his-tag.

DNA sequence and the corresponding translated peptide sequence is shown in Supplementary Fig. 1. The recombinant plasmid pHT43-SF-P was introduced into *B. subtilis* strains by electroporation. Recombinant *B. subtilis* clones were identified by PCR and the results are presented in Fig. 1A. The results showed that all single clones were positive clones with the correctly sized 543 bp target band (Fig. 1A). These results indicated that the target genes successfully recombined into pHT43, as anticipated.

After harvesting the culture medium, the proteins were analyzed by SDS-PAGE and western blot (Fig. 1B, C). The results showed that the target protein was expressed by the WB800N strain. The optimal conditions of expression were: 30 °C, 4 h, and 1 mM isopropyl *β*-d-1-thiogalactopyranoside (IPTG). The target protein was further isolated and purified by affinity Ni-Charged resin chromatography with a final purity of over 95%. SDS-PAGE displayed a clear single target protein band (Fig. 1D). The band of the target protein in the SDS-PAGE was excised and then submitted to in-gel digestion with trypsin. The amino acid sequence of the target protein was carried out using nano liquid chromatography-tandem mass spectrometry (Nano LC-MS/MS; Fig. 1E). The sequence coverage of the target protein was analyzed by MASCOT software analysis, and the sequence coverage was 90.86%. Therefore, through PCR, western blot, and Nano LC-MS/MS, it was demonstrated that the target protein (rsfAFP) was successfully constructed in the *B. subtilis* WB800N expression system.

### Antifreeze activities of rsfAFP

**Fig. 2:**
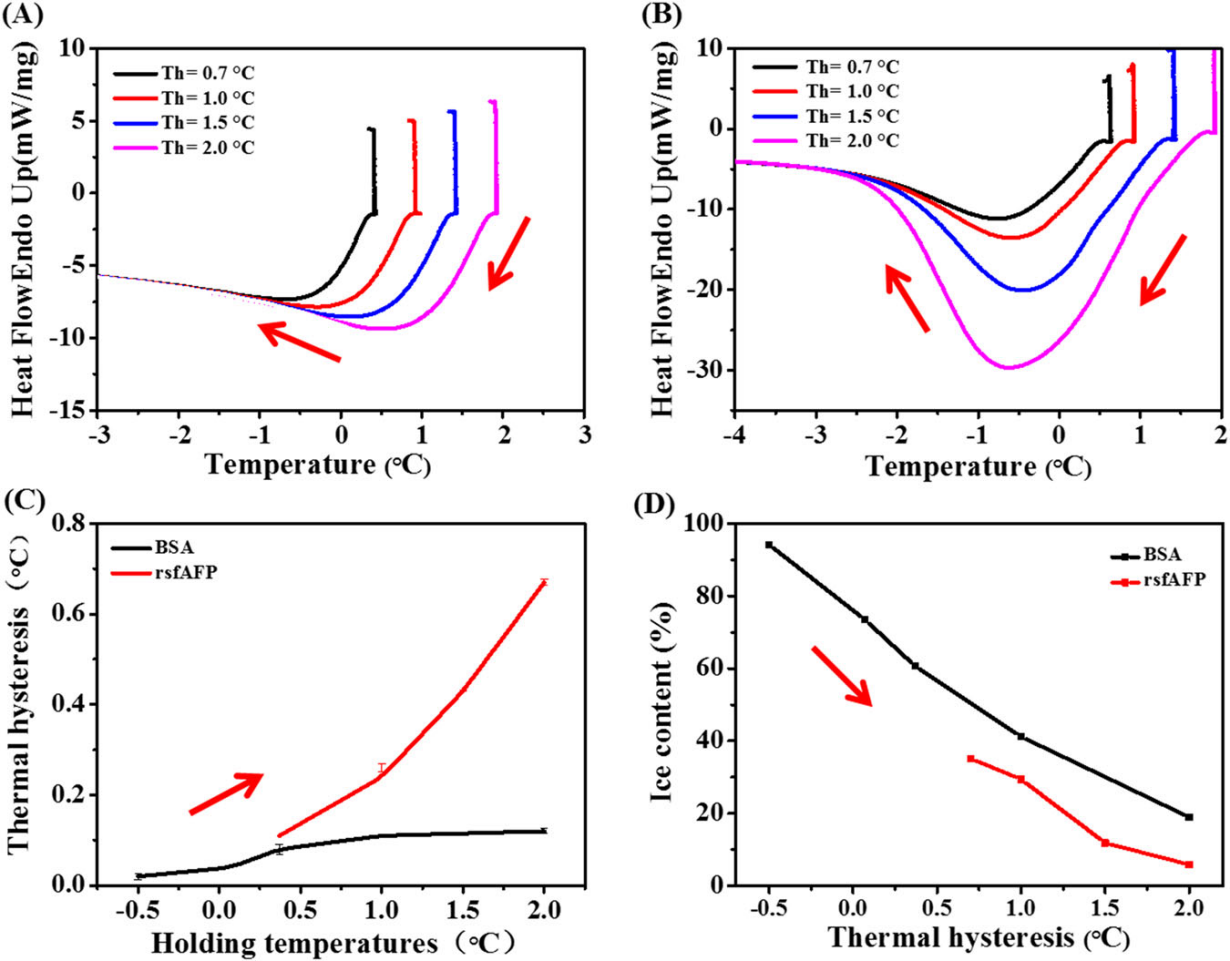
Antifreeze activities of rsfAFP. The DSC thermogram of the freezing and melting processes at different holding temperatures for **A** BSA and **B** rsfAFP. **C** The thermal hysteresis activity of BSA and rsfAFP at different holding temperatures. **D** The ice content of BSA and rsfAFP at different thermal hysteresis. Data points represent mean values calculated from two separate experiments.

AFPs are unique molecules that can bind at the growth interface of ice and are active on the ice-water interface. This property of AFPs mainly depends on their THA and IRI ability^26^. TH is due to the adhesion of AFPs on the ice surface, increasing the growth curvature of the ice crystals. This results in an increase in the vapor pressure of the ice-water interface layer, requiring greater energy for the ice crystal growth. THA is the gap between the freezing and melting points of a solution, and it is an important parameter to evaluate the activity of AFPs. Compared with the standard proteins (bovine serum albumin (BSA)) in the control groups, rsfAFP showed a significant TH exothermic peak (Fig. 2A, B). Through integral calculation, it was found that under the same holding temperature, the THA of rsfAFP increases significantly compared to BSA (Fig. 2C). Although the THA of rsfAFP is not excellent, the ice crystal content was significantly reduced after the addition of rsfAFP (Fig. 2D).

**Fig. 3:**
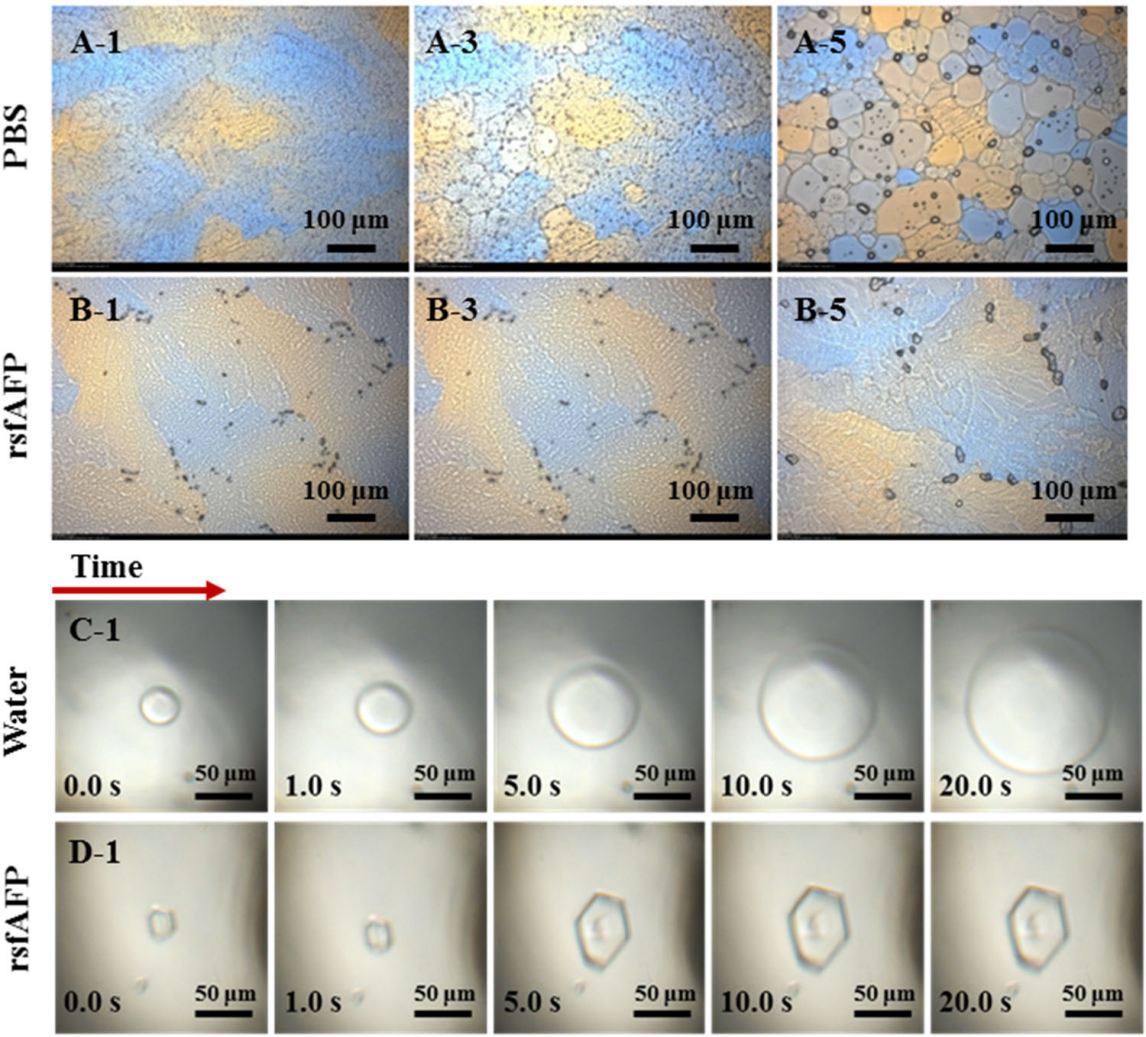
Optical images show the completely different growth behaviors and shapes of ice crystals with or without rsfAFP. **A**, **B** The effects of rsfAFP on ice recrystallization in PBS (50 mM NaH_2_PO_4_, 300 mM NaCl, pH 8.0) after different thermal cycles between −14 and −12 °C. **A-1**, **A-3**, and **A-5** show the ice crystal growth without rsfAFP before, after 3 cycles and 5 cycles, respectively. **B-1**, **B-3**, and **B-5** show the ice crystal growth with rsfAFP (0.1 mg/mL) before, after 3 cycles and 5 cycles, respectively. The morphology and growth rate of a single ice crystal without (**C**) or with (**D**) addition of 0.1 mg/mL rsfAFP in ultrapure water.

The frost-related damage of microorganisms during storage under freezing conditions is mainly caused by the recrystallization and thawing process^2,27^. Therefore, compared with THA, the IRI ability of AFPs is more important for frozen products during storage. The preferential adsorption of AFPs on the growth interface of ice to control the ice growth is widely accepted as conferring them their IRI ability and functional role in protecting organisms from freezing damage^14,27–29^. The IRI of rsfAFP was conducted via a polarized light microscope with a cold stage, and the results are shown in Fig. 3. The ice crystals in the negative groups grew significantly, circular in shape after five freeze-thaw cycles (Fig. 3A). It is worth noting that the ice crystal size of the samples with rsfAFP was significantly smaller than that of the negative group (Fig. 3B). These results indicated that there may be a specific interaction between rsfAFP and ice, inhibiting the recrystallization of ice crystals.

To demonstrate that rsfAFP can bind to the growth interface of ice crystals, the growth morphology of a single ice crystal was investigated using a nanoliter osmometer. As illustrated in Fig. 3C, in ultrapure water, ice is shaped like a flat disk and grows so rapidly that it takes only 20 s for the ice to fill the entire field of observation. Interestingly, the growth rate of ice crystals was significantly inhibited in rsfAFP aqueous dispersion, and the morphology of ice was significantly isomerized (Fig. 3D). The hexagonal shape of the ice is attributed to the fact that rsfAFP binds to the prism plane of ice^30–32^. The identification of antifreeze activity was demonstrated by the ability of rsfAFP to significantly regulate ice crystals.

### rsfAFP cytoprotection affects the physiological functions of *S. thermophilus* under freezing stress

**Fig. 4:**
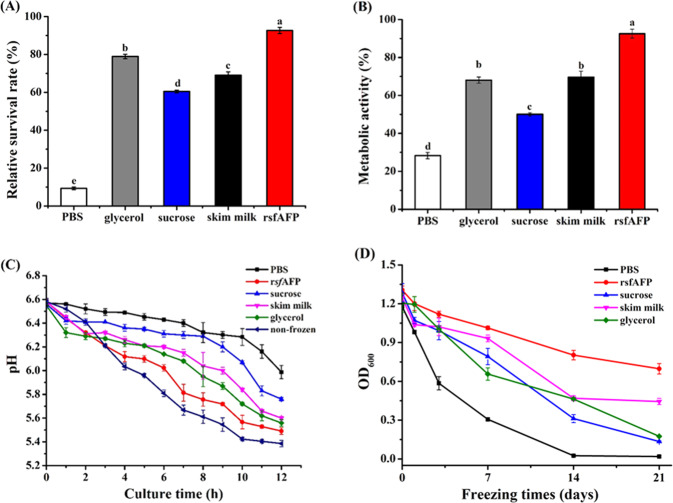
Cryoprotective effect of rsfAFP on *S. thermophilus*. **A** The survival rate of *S. thermophilus* after freezing at −20 °C for 24 h and 2 freezing–thawing cycles with various cryoprotectants. **B** *S. thermophilus* metabolic activity; **C** acid production of *S. thermophilus* with different cryoprotectants; **D** *S. thermophilus* growth with various cryoprotectants at different freezing times. The concentration of rsfAFP was 0.1 mg/mL. 20 mM PBS was used as a negative control; 15% glycerol, 1.0 mg/mL sucrose, and 1.0 mg/mL skim milk were used as the positive controls.

To evaluate the feasibility of rsfAFP as a substitute for current commercial cryoprotectants, a series of experiments were undertaken on the cryoprotective of rsfAFP on *S. thermophilus*. Different cryoprotectants were added to *S. thermophilus* and then exposed to freeze–thaw cycling models. The physiological functions of *S. thermophilus* with different cryoprotective formulations after freezing stress are shown in Fig. 4.

The survival rate of cryopreserved *S. thermophilus* thawed at 37 °C with different cryoprotectants is shown in Fig. 4A. The survival rate of *S. thermophilus* with 0.1 mg/mL rsfAFP was greatly increased from 8.86% to 93.21%, which was much higher than that observed when 1.0 mg/mL sucrose (60.71%), 1.0 mg/mL skim milk (65.39%), and 15% glycerol (79.80%) were used as cryoprotectants. During the freezing process, the extracellular water was first freezing into ice. The pressure difference between the inside and outside caused dehydration of the cell, increasing the intracellular electrolyte concentration. This elevated intracellular solute led to the denaturation or inactivation of sensitive enzymes as well as certain proteins, affecting the metabolic activity of cells. The metabolic vitality of *S. thermophilus* with different cryoprotectants is shown in Fig. 4B. The metabolic activity of the phosphate buffer solution (PBS), sucrose, skim milk, glycerol, and rsfAFP groups were 28.34, 50.04, 62.65, 71.06, and 82.54%, respectively. These results showed that the metabolic activity of *S. thermophilus* in the rsfAFP group was greatly improved when compared with the commercial cryoprotectant groups.

The acid production capacity is related to the metabolic activity of *S. thermophilus* cells, so the cryoprotective effect of different cryoprotectants on bacterial cells can be reflected by measuring the acid production. The acid production and the growing stability of *S. thermophilus* with different cryoprotectants after freezing stress are shown in Fig. 4C, D, respectively. Consistent with the trends for the survival rate, the acid production of rsfAFP group was significantly higher than that in commercial cryoprotectant groups. In addition, rsfAFP added to *S. thermophilus* significantly improved the freezing stability. After 14 days under freezing conditions, the survival rate in the PBS group decreased by 97.88%. The survival rate in the glycerol group, the most commonly used commercial cryoprotectant (glycerol group), still decreased by 61.57%. However, the survival rate of the rsfAFP group decreased only by 38.50% under the same storage conditions.

### Micromorphological characteristics of *S. thermophilus* cells

**Fig. 5:**
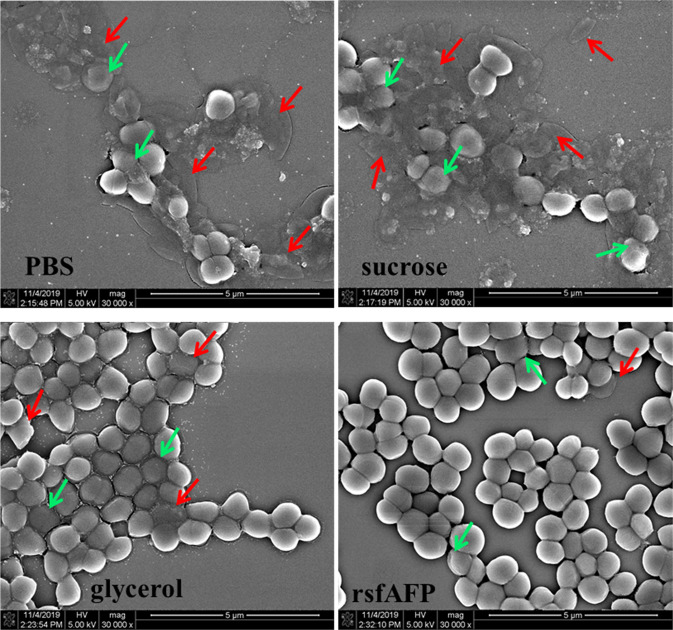
SEM images of *S. thermophilus* after freezing at −20 °C for 24 h and 2 freezing–thawing cycles with different cryoprotectants. The concentration of rsfAFP was 0.1 mg/mL. 20 mM PBS was used as s negative control; 15% glycerol, 1.0 mg/mL sucrose were used as the positive controls. Green arrow: damaged cells; red arrow: ruptured cells.

SEM observations revealed that the *S. thermophilus* cells without cryoprotectants were significantly damaged during frozen storage, and most of the cells had no intact cellular structure after freezing stress treatment. In addition, due to the rupture of the cell structure, the contents of *S. thermophilus* cells leaked out and deposited on the surface of the cells, making the cells adhere to each other without clear boundaries (Fig. 5). The cellular morphology in the two commercial cryoprotectants groups (sucrose and glycerol) was significantly improved when compared to the negative control group, but still showed varying degrees of damage. As anticipated, under the protection of rsfAFP, the cells were full, round and still presented a complete cell structure. Mechanical stress damage to *S. thermophilus* cells caused by the formation of big ice crystals during freezing storage is the main culprit for cell rupture. Previous studies on cell viability found that cell survival rate decreased after cryopreservation, likely caused by mechanical damage, and resulted in the inability of cells to fully recover. Therefore, rsfAFP can protect cells from the mechanical stress damage caused by big ice crystals during freezing storage by inhibiting the growth of ice crystals.

### Fourier transform infrared (FTIR) spectroscopic analysis

There is normally a lot of water on the surface of the cell membrane. The hydration layer formed between the polar head of the phospholipid and the water molecules around the cell membrane helps maintain the fluidity of the cell membrane. During the freezing or freeze-drying process, the hydration layer is destroyed, causing the phospholipid bilayer to change from a disordered liquid crystal phase to a rigid and ordered gel phase^33^. This change can cause serious damage to the cell membrane. Since lecithin is one of the important components that make up the specific structure of cell membranes, the interaction between rsfAFP and cell membrane can be simulated by measuring the interaction between rsfAFP and lecithin, allowing to explore the cryoprotective mechanism of rsfAFP on the cell membrane.

**Fig. 6:**
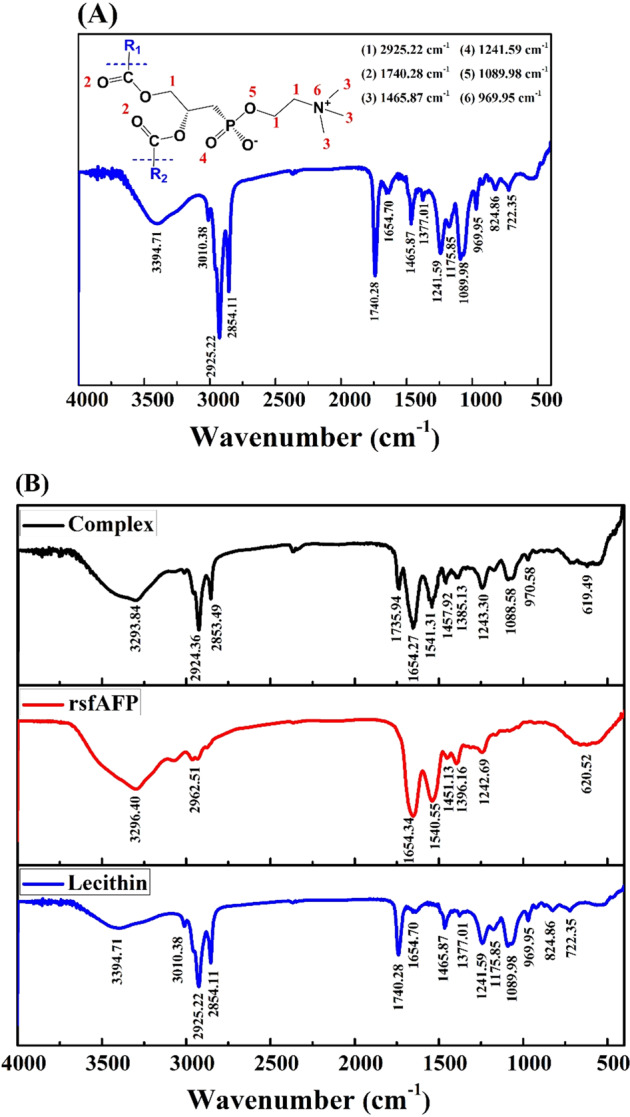
Fourier transform infrared (FTIR) spectroscopic analysis. **A** Characteristic FTIR spectra of egg yolk lecithin head; **B** FTIR spectra of egg yolk lecithin, rsfAFP, and their mixture.

The FTIR spectra of egg yolk lecithin head groups have distinct bands in the six-spectrum region (Fig. 6). These bands were 2925.22 cm^−1^, 1740.28 cm^−1^, 1465.87 cm^−1^, 1241.59 cm^−1^, 1089.98 cm^−1^, and 969.95 cm^−1^, corresponding to the egg yolk lecithin -CH_2_ stretching vibration, symmetric C=O stretching vibration, -CH_3_ antisymmetric deformation vibration, symmetric P=O stretching vibration, asymmetric P-O-C stretching vibration, and C-C-N^+^ stretching vibration, respectively^34,35^. Through the FTIR spectra analysis, it was found that the presence of rsfAFP changed the characteristic spectrum of lecithin head groups (Fig. 6). The wavenumbers of 1241.80 cm^−1^ and 969.95 cm^−1^ show redshift, indicating that the P=O group and C-C-N^+^ group of lecithin are unstable, and transfer to high wavenumber after combining with rsfAFP. In other words, the energy required for vibration after binding becomes larger and the group becomes more stable.

The hydrogen bonds between the polar head of the phospholipids and the hydroxyl groups of the AFP may prevent the phospholipid heads from approaching each other, resulting in a reduction of the vitrification phase transition temperature of the phospholipids, and maintaining the cell membrane fluidity^5,33,36^. Since rsfAFP is rich in hydroxyl groups, it can be speculated that the change in the characteristic band of egg yolk lecithin may be due to the formation of hydrogen bonds between the hydroxyl groups in the rsfAFP and the phospholipid head groups.

### In situ observation of *S. thermophilus* and ice crystals via Cryo-TEM

**Fig. 7:**
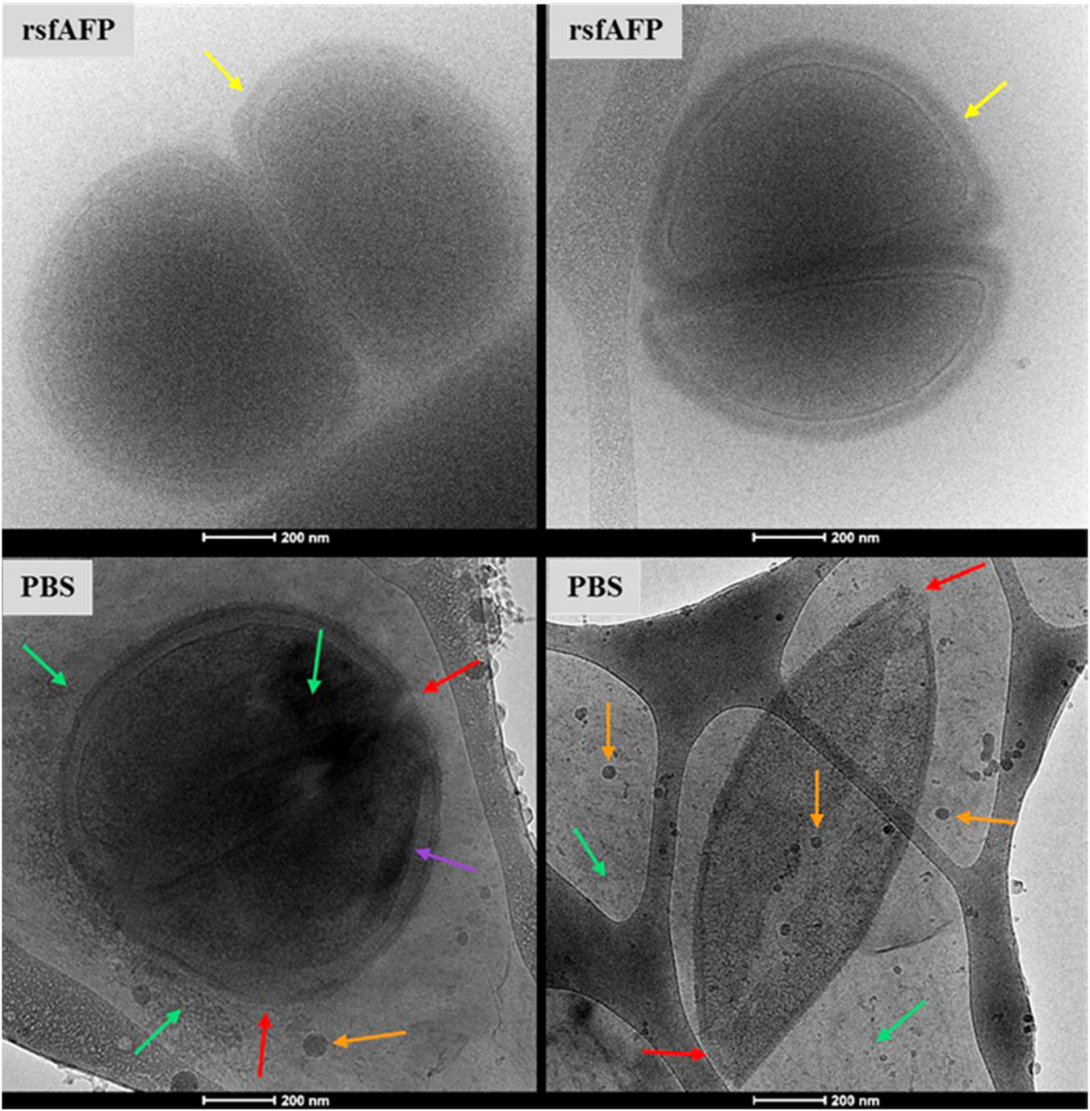
The microscopic states of ice crystals and *S. thermophilus* cells were observed by Cryo-TEM. The blurred background represents the ice crystal (green arrow); the cell wall of *S. thermophilus* ruptured (red arrow); the cell membrane shrinkage (purple arrow); the rsfAFP wrapped around *S. thermophilus* (yellow arrows); vesicles formed by bacterial rupture (orange arrows). The concentration of rsfAPP used for the cryoprotective measurement was 0.1 mg/mL, and 20 mM PBS was used as a negative control.

In order to further investigate the mechanism of action of rsfAFP on *S. thermophilus* cells, we conducted experiments with isolated *S. thermophilus* cells incubated with or without rsfAFP. These *S. thermophilus* cells were observed using Cryo-TEM. As shown in Fig. 7, cells without rsfAFP showed significant ruptures of the cell walls, shrinkage, and cellular deformations. In addition, a large number of ice crystals and vesicle-like particles were observed everywhere inside and outside the cells in the PBS control group, whereas the easily recognizable cytoskeleton and typical cell structure were clearly observed in the 0.1 mg/mL rsfAFP-treated cells. These results were consistent with those observed by SEM. Furthermore, the content in ice crystals around the cells treated with rsfAFP was obviously decreased, and the cytoderm in the rsfAFP group was significantly thicker than in the control group, giving the impression that a layer of material was wrapped around the cytoderm. Therefore, we speculated that rsfAFP acted by reducing the formation of ice crystals, therefore limiting the mechanical damage of ice crystals caused to cells. On the other hand, rsfAFP may act as a cellular scaffold wrapped around the outer layer of *S. thermophilus* cells, thus reducing the mechanical damage caused by ice crystals under freezing stress.

## Discussion

Compared to the traditional organic cryoprotectants (DMSO, glycerol, ethylene glycol, etc.), AFPs, with the advantages of less toxicity and non-cell membrane penetration, are becoming the most promising cryoprotectants for the cryopreservation of cells^37^. Although AFPs can more effectively regulate ice crystals than current cryoprotectants at low concentrations, a deeper understanding of their mechanisms of cryopreservation of cells is required^27^.

The cryopreserve effect of AFPs on organisms comes from the non-colligative reduction of AFPs, which inhibits ice crystal recrystallization. Numerous studies have shown that AFPs are specifically adsorbed at the growth interface of ice crystals to increase the growth curvature of ice and increase the vapor pressure of the ice-water interface layer, thereby lowering its freezing point^27,32,38^. The regulation effect of insect AFPs on ice crystals conforms to the “Janus” effect, which includes non-ice-binding faces (NIBF) and ice-binding faces (IBF)^39^. The IBF is attributed to the orderly arrangement of methyl and carboxyl groups on AFPs to form an ice-like hydration layer. The NIBF is a disorderly arrangement of a large number of charged side chains and hydrophobic side chains, and the interface water on the IBF is disordered. During the freezing process, the IBF of AFPs will be adsorbed on the growth interface of ice crystals, and the NIBF is exposed to water, disrupting the orderly arrangement of water at the interface and inhibiting the formation of ice nucleus. The IBF and the NIBF work together to inhibit the growth of ice crystals^31,39^. Snow flea antifreeze protein has a Polyproline type II Helix conformation^19^, which also includes IBF and NIBF (Supplementary Fig. 2). rsfAFP has significant THA (Fig. 2) and recrystallization inhibition ability (Fig. 3). In addition, by observing the morphology of single ice crystals, we showed that rsfAFP can significantly change the shape of the ice nucleus and inhibit the growth of ice crystals (Fig. 3D). The above experiments demonstrated that rsfAFP can be adsorbed on the growth interface of ice crystals. However, the molecular mechanism of rsfAFP on the regulation of ice crystals is still unclear.

Many studies have shown that the abundant hydroxyl groups in proteins/peptides can be combined through hydrogen bonds with the head groups of the phospholipid bilayer to maintain the fluidity of the cell membrane^5,36,40^. Through FTIR analysis, it was found that rsfAFP can interact with the phospholipid head group. Therefore, we believe that rsfAFP can bind to the phospholipid bilayer head group on the cell membrane through hydrogen bonds and protect the cell membrane structure during freezing. Combined with the Janus effect of rsfAFP, we speculate that the cryoprotective mechanism of rsfAFP on cells during freezing and freezing storage is demonstrated in Supplementary Fig. 3. The IBF of rsfAFP combines with ice crystals, and the NIBF disrupts the orderly arrangement of interfacial water and inhibits the formation of the ice nucleus. On the other hand, the excess hydroxyl groups rich in rsfAFP combined with the cell membrane phospholipid molecule head group through hydrogen bonds maintain the cell membrane fluidity and prevent ice crystals from damaging the cell membrane.

To verify the above assumption, rsfAFP and cells were observed in situ in the frozen state by Cryo-TEM. As shown in Fig. 7, many ice crystals appeared in the control group (without rsfAFP), and the cell structure was obviously broken. In addition, a large number of vesicle-like particles were formed around the cells in the control group. This is most likely due to the rupture of the cell wall caused by ice crystals, causing some damage to the cell membrane, and the increased intracellular mechanical stress causing the cells to burst and form vesicles^41^. Interestingly, no ice crystals were formed around the cells in the rsfAFP group, the cell environment remained in a glass state, and the cell structure was intact. It can be seen from the Cryo-TEM (Fig. 7) that a layer of species (yellow arrows) is wrapped in the extracellular text of the rsfAFP group, which demonstrated that rsfAFP is wrapped outside the cell membrane. These findings are consistent with the previous experiments related to the regulation effect of rsfAFP on ice crystals (Fig. 3) and the protection of cells (Fig. 4). Furthermore, since rsfAFP has a large molecular weight and cannot penetrate into the cell through the cell membrane, this peptide has a higher safety profile than small organic molecules such as glycerol^37^.

In summary, this study reported the first expression of rsfAFP using *B. subtilis*. The rsfAFP presented significant THA and recrystallization inhibition ability. Interestingly, rsfAFP can be adsorbed to the surface of the ice nucleus and change the growth trajectory of the ice nucleus to inhibit the growth of the ice crystals. Moreover, the viability, acid-producing ability, and freezing stability were significantly improved, while the decline of cellular metabolism activity of *S. thermophilus* was delayed with rsfAFP. Cryo-TEM results showed that rsfAFP can inhibit recrystallization and bind to cell membranes. FTIR analysis found that rsfAFP may bind to the head group of lecithin through hydrogen bonds. Collectively, these findings indicate that the cryoprotective mechanism of rsfAFP on cells is the interference with the cryogenic degeneration of cells through the synergistic effect of regulating ice crystals and maintaining the cell structure. These results highlight the potential value in food storage of rsfAFP as a novel cryoprotectant for LAB and frozen food to reduce the damage caused by ice crystals during freezing or thawing.

## Methods

### Materials and reagents

*B. subtilis* WB800N and pHT43 plasmid cloning vector were purchased from AtaGenix (Wuhan, China). The restricted endonuclease BamHI/XbaI and DNA purification kit were purchased from SanGon Biotech Co., Ltd (Shanghai, China). *S. thermophilus* used to investigate the damage caused by freezing stress was obtained from Shanghai Jiao Tong University (Shanghai, China). Other analytical reagents used in this work were purchased from Sigma-Aldrich, St, Louis, MO, USA.

### Construction of recombinant *B. subtilis* WB800N

rsfAFP is a peptide consisting of two repeats sequences of 6.5 kDa snow flea AFP^18^, which includes 175 amino acids. The nucleotide sequence was optimized according to *B. subtilis* WB800N codon preference, and the cDNA sequence encoding for rsfAFP was synthesized by Kingsley Biotech. Co., LTD. (Nanjing, China). This rsfAFP gene was double-digested with BamHI (ggatcc) and XbaI (tctaga) and cloned into pHT43 vector, which was renamed as pHT43-SF-P, and transformed into WB800N by electroporation. The pHT43-SF-P plasmid was extracted from WB800N and confirmed by PCR.

### Expression of pHT43*-*SF-P

WB800N competent cells (previously stored in a glycerin tube) were sub-inoculating in *B. subtilis* medium (5 mL) for 12 h at 30 °C, and then transferred to 600 mL of *B. subtilis* medium supplemented with erythromycin resistance (the final concentration is 50 μg/mL) for 3 h at 37 °C. When the OD_600_ reached 0.8–1.0, the medium was induced with 1 mM IPTG at 37 °C for 4 h. Cells were collected by centrifugation (3000 × *g*, 4 °C, 20 min), lysed in native buffer, centrifuged (C1) and the native protein extract recovered in the supernatant. The pellet harvested from C1 was resuspended in denaturing buffer (Urea 8 M) and centrifuged (3000 × *g*, 4 °C, 20 min). The supernatant contained the denatured protein extract, semi-purified from the culture medium. The proteins extracted from the native buffer, denaturing buffer and culture medium were analyzed by Tricine-SDS-PAGE and western blot using methods previously established by Zhang et al.^22^.

### Purification and identification of rsfAFP

The recombinant target protein was purified by affinity versus His-tag on Ni^2+^ resin. Briefly, a slurry of affinity versus His-tag was packed in a Ni^2+^ resin column (10 × 2.5 cm) and equilibrated with 5-column volume of equilibrating buffer and TBS buffer (20 mM Tris-HCl, 150 mM NaCl, pH 7.5). After harvesting the culture medium, 200 mL was loaded into the column. The column was washed 3 times with TBS buffer with 0, 30, and 50 mM imidazole, and the fractions were identified as W1, W2, and W3, respectively. The bound proteins were eluted using gradient elution with the same buffer containing 200 mM and 400 mM imidazole at a flow rate of 0.5 mL/min, the recovered fractions labeled E1 and E2, respectively. The eluate obtained from the culture medium was analyzed by SDS-PAGE. Briefly, 4 μL of the eluate was mixed with an equal volume of 2× sample buffer solution (6.86 M urea, 4.29% SDS, 300 mM DTT and 43 mM Tris-HCl, pH 6.8), and then incubated at 95 °C for 5 min. The samples were then quantitatively by SDS-PAGE grayscale analysis.

Nano LC-MS/MS was applied to identify the rsfAFP. The rsfAFP band in the SDS-PAGE was excised and then submitted to in-gel digestion with trypsin. The peptide sequencing was carried out and its molecular weight was measured using Nano LC-MS/MS. The detailed protocol for Nano LC-MS/MS analysis was as described by Liu et al.^42^. A Mascot MS/MS Ion Search was conducted with MS/MS data from Swiss-Prot and UniProt-Avena-1 databases.

### THA measurement

The THA of rsfAFP was evaluated with a 8500 DSC with Proteus Thermal Analysis software (PerkinElmer, USA), the detailed procedures having been previously described^43^.

### IRI assay

IRI activities of rsfAFP were determined with a polarizing microscope equipped with an optical CCD camera (Roper Scientific, USA) using a cold stage (Linkam, UK) with minor modifications from the previously described methods^44–46^. Briefly, a small drop (3 μL) of pH 7.4 TBS buffer (20 mM Tris-HCl, 150 mM NaCl) with or without rsfAFP (0.1 mg/mL) was placed between two circular glass coverslips. The coverslips were placed in the chamber of the cooling stage, and then rapidly cooled from room temperature to −50 °С at a rate of −10 °C/min and held for 5 min. The microscopic images of a sample at −50 °С were recorded through CCD camera, and then gradually the temperature was increased from −50 °С to −14 °С at a rate of 5 °C/min. The sample was then held at −14 °C for 1 min and the temperature increased to −12 °C at a rate of 1 °С/min. Afterward, a cycle of temperatures between −14 °C and −12 °С at a rate of 1 °C/min was performed 5 times, and the microscopic images were recorded for each sample.

### Nanoliter osmometer experiments

The morphology and growth rate of a single ice crystal were observed using a nanoliter osmometer with a stereomicroscope. The detailed protocol for the Nanoliter osmometer experiments was provided in the method described by Geng et al.^47^. Briefly, 0.5 nL of rsfAFP (0.1 mg/mL) was injected into a temperature-controlled sample holder full of silicone oil using a microsyringe and quickly frozen to −20 °C, and then slowly warmed to the melting temperature (*T*_m_). Once a single ice crystal appeared, the temperature was slowly decreased to the freezing temperature (*T*_f_) at which ice started to grow. The temperature was controlled within a margin of error of 0.01 °C, and throughout, the manipulations were protected by nitrogen (99.99%, 25 °C) to prevent air liquefaction to form a condensate. The evolution of an ice crystal was recorded by an optical CCD camera.

### Cryoprotective effect of rsfAFP on *S. thermophilus*

#### Freezing and thawing protocol

Briefly, *S. thermophilus* was previously sub-cultured in 20 mL of M17 broth (1%, v/v) at 200 rpm and 37 °C for 18 h, followed by inoculation in 20 mL of M17 broth (1%, v/v) and culture at 200 rpm 37 °C for 4 h until the cell density OD_600_ reached 1.0. Cells were harvested during the mid-log growth phase by centrifugation (6000 rpm for 10 min at 4 °C) and washed twice with 20 mM PBS (pH 7.4). The population of *S. thermophilus* in the suspension was approximately 10^8^ CFU/mL. To determine whether antifreeze agents effectively played a cryoprotective role in *S. thermophilus* after cold stress, the bacterial culture was diluted in PBS (pH 7.4) with 10 folds gradient dilution. 30 μL of diluted cells were added to 270 μL rsfAFP (0.1 mg/mL) in a 1.5 mL cryovials. Commercial cryoprotectants, including 1.0 mg/mL sucrose, 1.0 mg/mL skim milk and 15% glycerol (v/v), were used as positive groups. The phosphatic buffer solution (20 mM, pH 7.40) was used as a negative group. Aliquots (0.3 mL) of each bacterial suspension were frozen at -20 °C for 24 h and samples were subjected to 2 cycles of freeze-thaw in a 2-hour interval.

#### *S. thermophilus* cell viability assay

The viability of the *S. thermophilus* cells in each sample after cold stress were analyzed using the OD_600_ rapid detection according to the methods of Chen et al.^5^. The survival rate was expressed as OD_A_/OD_B_ × 100, where OD_A_ was the number of viable cells after freezing stress, and OD_B_ was the number of viable cells before freezing.

#### Acidifying activity measurement

The acidifying activity of *S. thermophilus* in each sample before and after cold stress was evaluated according to the method described by Meneghel et al.^33^.

#### Metabolic activity analysis

The metabolic activity of *S. thermophilus* was investigated according to Chen et al.^5^ using INT Analysis Kits.

### Scanning electron microscopy

The cells of *S. thermophilus* were freeze-dried using a lyophilizer (LGJ-18, Songyuan, Beijing, China) using a freezing/thawing protocol with or without cryoprotectant. The microscopy of *S. thermophilus* was performed by SEM (Nova Nano SEM 230, FEI Company, USA) after a small amount of gold was plated onto the samples.

### FTIR spectroscopy

The interactions between rsfAFP and the cell membrane were analyzed using egg yolk lecithin as a model according to the methods reported by Chen et al.^5^ Briefly, the samples (egg yolk lecithin, rsfAFP and their mixture) were mixed with potassium bromide 1 to 100. The spectra of each sample were analyzed using the OMNIC 8.2 software (Thermo Nicolet Co., Madison, WI, USA).

### In situ observation of *S. thermophilus* and ice crystals via Cryo-TEM

Cell solutions (5 μL) of *S. thermophilus* after freezing stress were dropped into grids (GiG, 200 mesh, carbon). Then vitrification was performed on the samples with a Vitrobot (FEI). These samples were observed under a Talos F200C G2 TEM (FEI). The images were captured with a CCD camera (Ceta 4 K*4K) under low dose conditions. A concentration of 0.1 mg/mL rsfAFP was used for the Cryo-TEM analysis and 20 mM PBS was used as a negative control.

### Statistical analysis

Data were analyzed by SPSS 17.0 (SPSS, Chicago, IL, USA) and reported as mean ± standard deviation from at least three to four independent experiments. Duncan’s multiple range test was used for statistical analysis and *P* < 0.05 was considered significant.

## Supplementary information

Supplementary Material

## Data Availability

We declare that all data related to this study are included in this paper and its Supplementary Information.

## References

[1] Mohan CO, Gunasekaran S, Ravishankar CN (2019). Chitosan-capped gold nanoparticles for indicating temperature abuse in frozen stored products. npj Sci. Food.

[2] Zhu Z, Zhou Q, Sun D-W (2019). Measuring and controlling ice crystallization in frozen foods: a review of recent developments. Trends Food Sci. Technol..

[3] Chen X, Wu J, Cai X, Wang S (2021). Production, structure–function relationships, mechanisms, and applications of antifreeze peptides. Compr. Rev. Food Sci. Food Saf..

[4] Zachariassen KE, Kristiansen E (2000). Ice nucleation and antinucleation in nature. Cryobiology.

[5] Chen X, Wu JH, Li L, Wang SY (2019). Cryoprotective activity and action mechanism of antifreeze peptides obtained from Tilapia scales on *Streptococcus thermophilus* during cold stress. J. Agr. Food Chem..

[6] Cao L (2016). Neofunctionalization of zona pellucida proteins enhances freeze-prevention in the eggs of Antarctic notothenioids. Nat. Commun..

[7] Hasan M, Fayter AER, Gibson MI (2018). Ice recrystallization inhibiting polymers enable glycerol-free cryopreservation of microorganisms. Biomacromolecules.

[8] Devries AL (1971). Glycoproteins as biological antifreeze agents in antarctic fishes. Science.

[9] Du L, Betti M (2016). Identification and evaluation of cryoprotective peptides from chicken collagen: ice-growth inhibition activity compared to that of type I antifreeze proteins in sucrose model systems. J. Agric. Food Chem..

[10] Kong LF, Qatran Al-Khdhairawi AA, Tejo BA (2020). Rational design of short antifreeze peptides derived from Rhagium inquisitor antifreeze protein. Biocatal. Agric. Biotechnol..

[11] Biggs CI (2017). Polymer mimics of biomacromolecular antifreezes. Nat. Commun..

[12] Mochizuki K, Molinero V (2018). Antifreeze glycoproteins bind reversibly to ice via hydrophobic groups. J. Am. Chem. Soc..

[13] Chen X, Li L, Yang FJ, Wu JH, Wang SY (2020). Effects of gelatin-based antifreeze peptides on cell viability and oxidant stress of *Streptococcus thermophilus* during cold stage. Food Chem. Toxicol..

[14] Kashyap P, Kumar S, Singh D (2020). Performance of antifreeze protein HrCHI4 from *Hippophae rhamnoides* in improving the structure and freshness of green beans upon cryopreservation. Food Chem..

[15] Liu M (2018). Comparative study on the cryoprotective effects of three recombinant antifreeze proteins from *Pichia pastoris* GS115 on hydrated gluten proteins during freezing. J. Agric. Food Chem..

[16] Chen X, Wu JH, Li L, Wang SY (2017). The cryoprotective effects of antifreeze peptides from pigskin collagen on texture properties and water mobility of frozen dough subjected to freeze–thaw cycles. Eur. Food Res. Technol..

[17] Graham LA, Davies PL (2005). Glycine-rich antifreeze proteins from snow fleas. Science.

[18] Pentelute BL (2008). X-ray structure of snow flea antifreeze protein determined by racemic crystallization of synthetic protein enantiomers. J. Am. Chem. Soc..

[19] Treviño MÁ (2018). The singular NMR fingerprint of a polyproline II helical bundle. J. Am. Chem. Soc..

[20] Lin F-H, Graham LA, Campbell RL, Davies PL (2007). Structural modeling of snow flea antifreeze protein. Biophys. J..

[21] Davies PL (2014). Ice-binding proteins: a remarkable diversity of structures for stopping and starting ice growth. Trends Biochem. Sci..

[22] Zhang (2018). Intracellular expression of antifreeze peptides in food grade *Lactococcus lactis* and evaluation of their cryoprotective activity. J. Food Sci..

[23] Todde G, Whitman C, Hovmöller S, Laaksonen A (2014). Induced ice melting by the snow flea antifreeze protein from molecular dynamics simulations. J. Phys. Chem. B.

[24] Bissoyi A (2019). Ice nucleation properties of ice-binding proteins from snow fleas. Biomolecules.

[25] Hong HA (2008). The safety of *Bacillus subtilis* and *Bacillus indicus* as food probiotics. J. Appl. Microbiol.

[26] Middleton AJ (2012). Antifreeze protein from freeze-tolerant grass has a beta-roll fold with an irregularly structured ice-binding site. J. Mol. Biol..

[27] Chen X (2020). Ice-binding proteins: a remarkable ice crystal regulator for frozen foods. Crit. Rev. Food Sci. Nutr..

[28] Song DH (2019). Cryoprotective effect of an antifreeze protein purified from *Tenebrio molitor* larvae on vegetables. Food Hydrocolloid.

[29] Bar Dolev M, Braslavsky I, Davies PL (2016). Ice-binding proteins and their function. Annu. Rev. Biochem..

[30] Bayer-Giraldi M (2018). Growth suppression of ice crystal basal face in the presence of a moderate ice-binding protein does not confer hyperactivity. Proc. Natl Acad. Sci. USA.

[31] Hudait A, Odendahl N, Qiu Y, Paesani F, Molinero V (2018). Ice-nucleating and antifreeze proteins recognize ice through a diversity of anchored clathrate and ice-like motifs. J. Am. Chem. Soc..

[32] Meister K, DeVries AL, Bakker HJ, Drori R (2018). Antifreeze glycoproteins bind irreversibly to ice. J. Am. Chem. Soc..

[33] Meneghel J, Passot S, Dupont S, Fonseca F (2017). Biophysical characterization of the *Lactobacillus delbrueckii* subsp. bulgaricus membrane during cold and osmotic stress and its relevance for cryopreservation. Appl. Microbiol. Biotechnol..

[34] Byler DM, Susi H (1986). Examination of the secondary structure of proteins by deconvolved FTIR spectra. Biopolymers.

[35] Tai K (2018). The effect of sterol derivatives on properties of soybean and egg yolk lecithin liposomes: stability, structure and membrane characteristics. Food Res. Int..

[36] Wang W, Chen M, Wu J, Wang S (2015). Hypothermia protection effect of antifreeze peptides from pigskin collagen on freeze-dried *Streptococcus thermophiles* and its possible action mechanism. LWT Food Sci. Technol..

[37] Białkowska A, Majewska E, Olczak A, Twarda-Clapa A (2020). Ice binding proteins: diverse biological roles and applications in different types of industry. Biomolecules.

[38] Hudait A, Qiu Y, Odendahl N, Molinero V (2019). Hydrogen-bonding and hydrophobic groups contribute equally to the binding of hyperactive antifreeze and ice-nucleating proteins to ice. J. Am. Chem. Soc..

[39] Liu K (2016). Janus effect of antifreeze proteins on ice nucleation. Proc. Natl Acad. Sci. USA.

[40] Allison SD, Chang B, Randolph TW, Carpenter JF (1999). Hydrogen bonding between sugar and protein is responsible for inhibition of dehydration-induced protein unfolding. Arch. Biochem. Biophys..

[41] Toyofuku M, Nomura N, Eberl L (2019). Types and origins of bacterial membrane vesicles. Nat. Rev. Microbiol..

[42] Liu H (2015). iTRAQ-based quantitative proteomic analysis of *Pseudomonas aeruginosa* SJTD-1: a global response to n-octadecane induced stress. J. Proteomics.

[43] Wu J (2015). Isolation and characterisation of sericin antifreeze peptides and molecular dynamics modelling of their ice-binding interaction. Food Chem..

[44] Li L (2017). Investigation of the physiochemical properties, cryoprotective activity and possible action mechanisms of sericin peptides derived from membrane separation. LWT Food Sci. Technol..

[45] Wang SY, Agyare K, Damodaran S (2009). Optimisation of hydrolysis conditions and fractionation of peptide cryoprotectants from gelatin hydrolysate. Food Chem..

[46] Damodaran S, Wang S (2017). Ice crystal growth inhibition by peptides from fish gelatin hydrolysate. Food Hydrocolloid.

[47] Geng H (2017). Graphene oxide restricts growth and recrystallization of ice crystals. Angew. Chem. Int. Ed..

