# Supplementary material for: Snow flea antifreeze peptide for cryopreservation of lactic acid bacteria
Source: NPJ Sci Food. 2022 Feb 3;6:10. doi: 10.1038/s41538-022-00128-4 (PMC8813996; doi:10.1038/s41538-022-00128-4)
Supplement: Supplementary file 1 — Supplementary Material [file 41538_2022_128_MOESM1_ESM.pdf]

Snow flea antifreeze peptide: An excellent antifreeze agent for cryopreservation of lactic acid bacteria

Xu Chen[1,2,†], Jinhong Wu[3,†], Xiaozhen Li[1], Fujia Yang[1,2], Jianlian Huang[4,5], Shaoyun Wang[2,*], Vincent Guyonnet[6]

[1] College of Biological Science and Engineering, Fuzhou University, Fuzhou, Fujian 350108, China

[2] College of Chemical Engineering, Fuzhou University, Fuzhou, Fujian 350108, China

[3] Department of Food Science and Engineering, School of Agriculture and Biology, Shanghai Jiao Tong University, Shanghai 200240, China

[4] Fujian Provincial Key Laboratory of Frozen Processed Aquatic Products, Xiamen 361022, China

[5] Fujian Anjoy Food Co. Ltd., Xiamen 361022, China

[6] FFI Consulting Ltd, 2488 Lyn Road, Brockville ON K6V 5T3, Canada

---

[†]Xu Chen and Jinhong Wu are contributed equal to this paper.

[*] **Corresponding author:**

Prof. Dr. Shaoyun Wang

College of Biological Science and Engineering, Fuzhou University, China

E-mail: shywang@fzu.edu.cn

Tel: +86-591-22866375. Fax: +86-591-22866278.

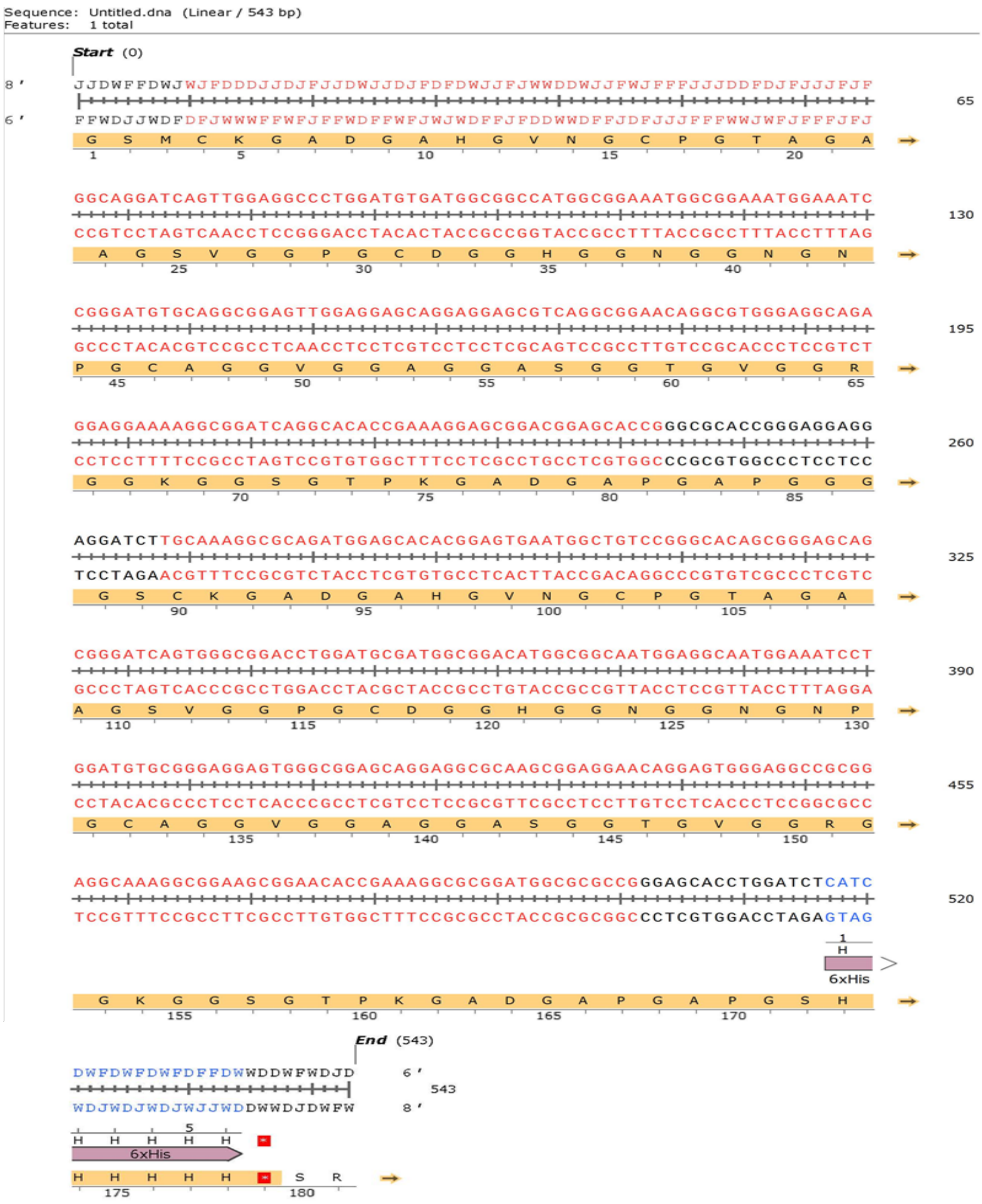

Supplementary Figure 1. **The amino acid and corresponding DNA sequences of rsfAFP**. The figure was created by SnapGene 4.1.9. DNA sequencing was performed using NCBI BLAST tools (https://blast.ncbi.nlm. nih.gov/Blast.cgi), which confirmed an exact sequence match.

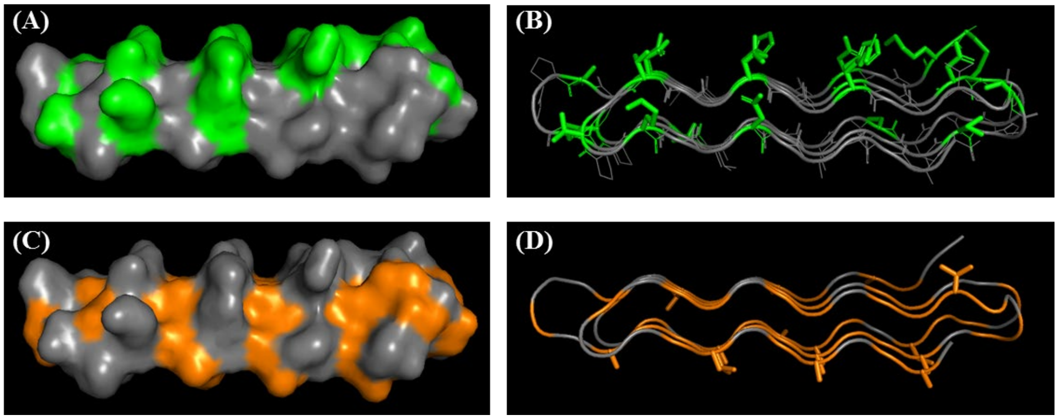

Supplementary Figure 2. **Spatial structure analysis of rsfAFP**. (A) and (B) are the ice-binding faces (IBF); (A) and (B) are the non-ice-binding faces (NIBF).

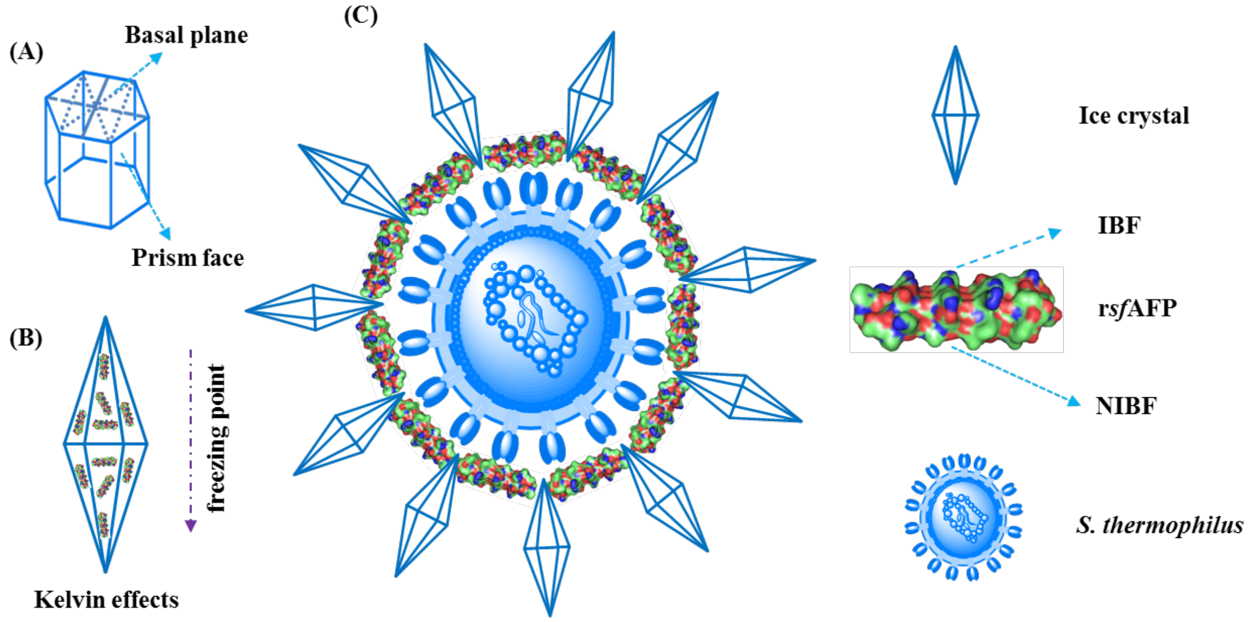

Supplementary Figure 3. **Cryoprotective mechanism of rsfAFP on *S. thermophilus* during freezing and frozen stage**. (A) Typical ice crystal morphology; (B) Interaction between rsfAFP and the prism surface changes the growth trajectory of ice crystals; (C) Models of rsfAFP interacting with ice crystals and *S. thermophilus*. IBF, Ice-binding face; NIBF, Non-ice-binding face.
